# Impact of alcohol use discontinuation on adipose tissue insulin resistance among Latino patients with and without underlying chronic liver disease

**DOI:** 10.1038/s41598-026-65801-0

**Published:** 2026-08-07

**Authors:** Shyam Patel, Albert Do, Amy Shui, Mandana Khalili

**Affiliations:** 1 Department of Medicine, Division of Gastroenterology and Hepatology, Kaiser Permanente San Francisco Medical Center, San Francisco, CA USA; 2 Department of Internal Medicine, Division of Gastroenterology and Hepatology, University of California, Davis, Sacramento, CA USA; 3 Department of Epidemiology and Biostatistics, University of California, San Francisco, San Francisco, CA USA; 4 Department of Medicine, Division of Gastroenterology and Hepatology, University of California San Francisco, San Francisco General Hospital, 1001 Potrero Avenue, Building 5, Suite 3D4, San Francisco, CA 94110 USA

**Keywords:** Hispanic, Hepatitis C, Health disparities, Socioeconomically disadvantaged populations, Cardiometabolic health, Diseases, Endocrinology, Gastroenterology, Genetics, Medical research

## Abstract

We evaluated the effect of short-term moderate alcohol discontinuation on adipose tissue insulin resistance (adipo-IR) and factors associated with its change in Latinos with and without hepatitis C virus (HCV). Nondiabetic Latino adults without (N = 17) or with (N = 7) HCV underwent clinical, laboratory, genetic, and metabolic testing. Metabolic testing was repeated after 6.5 weeks of moderate alcohol discontinuation. Adipo-IR was measured using a 2-step insulin suppression test. Multivariable linear regression, including HCV status, identified factors associated with change in adipo-IR. Median age was 50 years, 58% female, median BMI 26.3 kg/m². Genetic ancestry was 57% European, 36% Native American, and 3.5% African. CYP2E1 genotyping showed 14% were homozygous for [c2/c2] risk-allele. No significant changes in adipo-IR occurred after alcohol discontinuation. On multivariable regression, CYP2E1 homozygous risk-allele (vs. none, estimate 0.25 mmol/L, p < 0.001) and overweight/obesity (vs. normal weight, estimate 0.13 mmol/L, p = 0.02), but not HCV, were associated with change in adipo-IR following alcohol discontinuation. Although there was no significant overall change in adipo-IR following short-term moderate alcohol discontinuation, CYP2E1 homozygous risk-allele and overweight/obesity were associated with changes in adipo-IR. These exploratory findings suggest that host genetic and metabolic characteristics may influence metabolic responses to alcohol discontinuation and warrant validation in larger prospective studies.

## Introduction

Alcohol-associated liver disease (ALD) is a leading cause of chronic liver disease in the United States and disproportionately affects vulnerable populations, including racial and ethnic minorities^1,2^. ALD frequently coexists with other metabolic dysfunction-associated chronic liver diseases, particularly hepatitis C virus (HCV) infection^3^. Among patients with ALD, underlying insulin resistance (IR) is one of the strongest predictors of liver fibrosis severity and hepatic inflammation^4^. HCV infection itself promotes IR, and treatment of HCV has been shown to improve these metabolic disturbances^5,6^. Concomitant metabolic dysfunction and alcohol use has also been associated with higher all-cause mortality, largely due to the additional detrimental effects of alcohol^7^. Furthermore, Marti-Aguado et al. showed that even low to moderate alcohol consumption increases liver fibrosis risk among individuals with chronic liver disease and cardiometabolic risk factors^8^. However, the impact of discontinuing low to moderate alcohol use on metabolic parameters in chronic liver disease has not been well evaluated. Given the contribution of both alcohol and HCV to liver disease progression, understanding how modifiable factors, such as alcohol discontinuation, impact metabolic dysfunction is critical. While we have previously shown a relationship between short-term alcohol cessation and hepatic IR, but not muscle IR^9^ in a Latino cohort, its impact on adipose tissue insulin resistance (adipo-IR) is not clearly understood. Considering the disproportionate impact of liver disease in this at-risk population, we studied a cohort of non-diabetic Latinos with and without HCV and conducted comprehensive demographic, behavioral, clinical, and genetic profiling. This included genotyping for cytochrome P450 2E1 (*CYP2E1*) and patatin-like phospholipase domain-containing protein 3 (*PNPLA3*), two genetic variants highly prevalent in the Latino population and implicated in the progression of chronic liver disease^10–13^. We now extend our assessment to evaluating the impact of discontinuation of short-term moderate alcohol use on adipo-IR and identified factors associated with change in adipo-IR.

## Methods

### Study population

Non-diabetic Latino adults (≥ 18 years old) with and without chronic HCV who reported moderate alcohol consumption were enrolled and underwent study procedures and follow-up at the Zuckerberg San Francisco General Hospital from June 2013 through May 2015 as previously described^9^. In Uribe et al. study, hepatic and muscle IR was assessed and in our current study adipo-IR was evaluated using patient samples. Moderate alcohol use for women was defined as up to one drink per day (≤ 14 g of ethanol/day) and no more than 7 drinks per week (≤ 98 g of ethanol/week), and for men, up to two drinks per day (≤ 28 g of ethanol/day) and no more than 14 drinks per week (≤ 196 g of ethanol/week)^14^. Latino ethnicity was defined by self-report of Latino ethnicity of all four biologic grandparents and both parents^15,16^. Chronic HCV infection was confirmed by detectable HCV viral load. Exclusion criteria included a history of diabetes (based on fasting glucose ≥ 126 mg/dL, 120-minute glucose ≥ 200 mg/dL on oral glucose tolerance test (OGTT), prior diagnosis of diabetes or use of antidiabetic medications), human immunodeficiency virus infection, any known etiologies of liver disease other than HCV, and other medical conditions in which frequent blood draws are contraindicated (e.g., anemia and cardiovascular disease). HCV-infected individuals were excluded if they had cirrhosis (clinical or histologic), decompensated liver disease, or prior or current HCV therapy. All participants provided written informed consent. The study was approved by the Institutional Review Board (IRB) of the University of California, San Francisco and the Zuckerberg San Francisco General Hospital. The trial was prospectively registered at ClinicalTrials.gov (NCT01858012; registered May 17, 2013), and registration occurred prior to enrollment of the first participant.

### Study procedures

At an initial screening visit, the patients’ demographic and clinical history, and anthropometric measurements (height, weight, body mass index [BMI], and waist circumference) were taken, and laboratory evaluations performed. A thorough drinking history was obtained for each patient via face-to-face interview at each study visit. Recent drinking behavior over the prior 12 months was assessed across three domains: drinking levels (number of drinks per day and number of drinking days per week), patterns, and intake variability. All study participants were instructed to and agreed upon discontinuing alcohol use for the following six weeks.

### Metabolic testing

Metabolic testing was performed during a 2-day inpatient visit to the UCSF Clinical and Translational Science Institute’s Clinical Research Center as previously described both at baseline and six weeks following alcohol discontinuation^9,17^. During this stay, an OGTT (day 1) was performed. Based on OGTT results, participants who met diagnostic criteria for diabetes (120-minute glucose level ≥ 200 mg/dL) were excluded^18^.

On hospital day 2, the 240-minute 2-step insulin suppression test (IST) (low dose and high dose) was performed as previously described^9,17^. Adipo-IR was measured in each subject by assessment of degree of impairment of insulin-mediated adipose tissue lipolysis during the first step of the IST following a 12-hour overnight fast. Briefly, to suppress endogenous insulin secretion, subjects were infused with octreotide at the rate of 0.27 µg/[m²·min] during the entire 240-minute study duration. Infusion rates of insulin and glucose during the first stage were at 6 mU/[m²·min]. At 100 to 120 min, when steady state of the first stage of IST was reached, representing the basal state (plasma insulin concentrations of ~ 15 µU/mL), blood was drawn at 10-minute intervals for measurement of plasma insulin, glucose, free fatty acids (FFA), and glycerol concentrations. The mean of these values represents the insulin, FFA, and glycerol concentrations for each individual study participant. The steady state plasma FFA levels during the first step of IST represents adipo-IR^19^. The metabolic testing was then repeated following 6 weeks of alcohol discontinuation.

A complete follow-up on alcohol abstinence was assessed by self-report (weekly phone calls and reminders) and confirmed with random urine ethyl glucuronide testing (performed twice at least two weeks apart).

### Genetic testing

All study participants underwent genotyping to detect polymorphisms in the *CYP2E1* RsaI c2 and *PNPLA3* rs738409 (C/G) allele. *CYP2E1*, an alcohol-metabolizing enzyme with a relatively high frequency in Latino populations, increases risk for developing ALD^10,13,20^. *PNPLA3*, also more prevalent in Latinos, has been linked to the development of steatotic liver disease^11,12^. DNA was derived from whole blood samples using the QIAamp DNA Blood Mini Kit (QIAGEN Sciences, Germantown, MD). Genotyping was performed as previously reported^21^.

### Estimation of ancestry for Latino individuals

The Latino population is an admixed group assumed to be comprised of three ancestral populations: African, European, and Native American. To identify and control for the heterogeneity among Latino people from different origins, we estimated the proportion of global ancestry for each individual as previously described^10,17,20,22^. One hundred and four autosomal ancestry informative markers (AIMs) across the genome were used for the inference of individual ancestry proportions for our Latino participants (%African, %Native American, and %European heritage). These AIMs were selected because their allele frequencies vary between African, European, and Native American populations.

### Statistical analysis

Baseline characteristics of study patients were tabulated or reported using median (1st −3rd quartiles). P-values comparing HCV + to HCV- participants on continuous variables were obtained by Wilcoxon rank-sum tests, and for categorical variables they were obtained by Chi-squared tests. For comparison of parameters before and after alcohol cessation, Wilcoxon signed-rank tests were used. We used linear regression, reported with 95% confidence intervals (CI), to examine the associations of participant characteristics with changes in adipo-IR. Linear regression assumptions were assessed using quantile-quantile and residual plots and by testing the significance of the quadratic term for continuous risk factor variables. For multivariable modelling, HCV was included in the model and forward stepwise selection process was used to select additional variables one by one with p-value below 0.05 for entry in each step and stopping when no new variables had *p* < 0.05. We selected at most one other risk factor variable at a time, and each selection considered clinical judgment in addition to statistical significance. All potential risk factors considered for inclusion in the model were pre-defined by clinical knowledge. Hypothesis tests were two-sided, and the significance threshold was set to 0.05. All analyses were performed using the Statistical Analysis System version 9.4 (SAS Institute, Cary, NC).

## Results

### Cohort characteristics

Twenty-four Latino study participants (*N* = 17 without HCV, *N* = 7 with HCV) who drank alcohol moderately were included in the study. Metabolic evaluation was performed at baseline and after a median of 6.5 weeks (Q1-Q3 5.9–7.7 weeks) following alcohol discontinuation. The overall cohort characteristics were as follows: median age 50 years, 58% women, and median BMI 26.3 kg/m^2^. In our study population, the median percent of European, Native American, and African ancestry was 57.3, 36.0, and 3.5, respectively. While the proportion of African American ancestry was significantly higher (16% vs. 3.1%), the proportion of Native American ancestry (25% vs. 41%) was lower among HCV + vs. HCV- patients (both *p* < 0.05). SNP genotyping profiles for *CYP2E1* and *PNPLA3* were as follows: 77% [c1/c1], 9.1% heterozygous risk-allele [c1/c2], 14% homozygous risk-allele [c2/c2] and 25% [CC], 42% [CG], and 33% [GG]. As anticipated, HCV+ patients had significantly higher alanine transaminase (ALT) (median ALT 53.0 vs. 27.0 U/L) and aspartate aminotransferase (AST) levels (median AST 47.0 vs. 23.0 U/L) at baseline compared to HCV- patients (both *p* < 0.05). Moreover, HCV+ patients had lower total cholesterol (median total cholesterol 122.0 vs. 184.0 mg/dL) and low-density lipoprotein **(**LDL) (median LDL 49.0 vs. 109.0 mg/dL) levels at baseline compared to HCV- patients. At baseline, there was not a statistically significant difference in adipo-IR levels between HCV + vs. HCV- patients (0.4 vs. 0.3 mmol/L). Further comparison of metabolic parameters can be found in Table 1.

**Table 1:** Cohort characteristics, stratified by HCV status.

Characteristics	Total (*N* = 24)*	HCV+ (*N* = 7)*	HCV- (*N* = 17)*	*p*-value
Age [median (Q1,Q3)] years	50.0 (36.0, 55.0)	55.0 (51.0, 55.0)	41.0 (36.0, 53.0)	0.17
Female sex [*N* (%)]	14 (58)	4 (57)	10 (59)	0.94
BMI [median (Q1,Q3)] kg/m^2^	26.3 (24.6, 29.7)	23.4 (21.7, 27.8)	26.9 (25.9, 30.4)	0.11
Waist circumference [median (Q1,Q3)] cm	95.5 (79.4, 101.9)	95.0 (76.5, 101.8)	96.5 (80.6, 102.1)	0.57
Current smoker [*N* (%)]	3 (13)	1 (14)	2 (12)	0.87
% European ancestry [median (Q1,Q3)]	(*N* = 21)	(*N* = 5)	(*N* = 16)	0.30
	57.3 (47.4, 60.8)	57.8 (50.5, 72.2)	56.2 (41.8, 60.5)	–
% African ancestry [median (Q1,Q3)]	(*N* = 21)	(*N* = 5)	(*N* = 16)	**0.02**
	3.5 (0.8, 7.5)	16.3 (13.5, 17.3)	3.1 (0.7, 5.7)	
% Native American ancestry [median (Q1,Q3)]	(*N* = 21)	(*N* = 5)	(*N* = 16)	**0.01**
	36.0 (30.6, 47.2)	24.9 (24.3, 27.7)	41.0 (32.0, 54.6)	–
PNPLA3 [*N* (%)]	–	–	–	0.42
CC	6 (25)	3 (43)	3 (18)	–
CG	10 (42)	2 (29)	8 (47)	–
GG	8 (33)	2 (29)	6 (35)	–
CYP2E1 [*N* (%)]	(*N* = 22)	(*N* = 5)	(*N* = 17)	0.67
Reference (c1/c1)	17 (77)	1 (20)	2 (12)	–
Heterozygous (c1/c2)	2 (9.1)	0 (0.0)	2 (12)	–
Homozygous (c2/c2)	3 (14)	4 (80)	13 (76)	–
Fasting glucose [median (Q1,Q3)] (mg/dL)	94.4 (88.5, 104.4)	94.5 (94.3, 107.0)	93.0 (88.5, 98.3)	0.14
Fasting insulin [median (Q1,Q3)] (µU/mL)	11.6 (7.6, 18.3)	12.0 (7.3, 22.8)	11.3 (8.0, 17.1)	0.53
Fasting glycerol [median (Q1,Q3)] (mmol/L)	50.0 (37.0, 58.8)	50.5 (45.0, 73.0)	48.0 (36.5, 58.0)	0.50
Fasting FFA [median (Q1,Q3)] (mmol/L)	0.4 (0.3, 0.5)	0.4 (0.3, 0.5)	0.4 (0.3, 0.4)	0.53
Adipo-IR [median (Q1,Q3)] (mmol/L)	0.3 (0.1, 0.4)	0.4 (0.1, 0.5)	0.3 (0.2, 0.3)	0.66
Total cholesterol (mg/dL)	166.5 (140.5, 206.0)	122.0 (101.0, 165.0)	184.0 (161.0, 206.0)	**0.009**
HDL cholesterol (mg/dL)	47.5 (44.0, 61.5)	60.0 (44.0, 67.0)	46.0 (44.0, 61.0)	0.36
LDL cholesterol (mg/dL)	98.0 (66.0, 120.0)	49.0 (41.0, 91.0)	109.0 (92.0, 137.0)	**0.004**
Triglyceridies (mg/dL)	78.0 (61.5, 142.5)	64.0 (45.0, 97.0)	100.0 (64.0, 148.0)	0.29
AST [median (Q1,Q3)] units/L	24.5 (21.0, 40.0)	47.0 (25.0, 79.0)	23.0 (20.0, 30.0)	**0.01**
ALT [median (Q1,Q3)] units/L	28.0 (24.0, 35.0)	53.0 (27.0, 98.0)	27.0 (21.0, 29.0)	**0.02**

**Bold** indicates *p* < 0.05.

*Unless otherwise specified in the table.

### Changes in metabolic parameters following alcohol discontinuation

As depicted in Table 2, following six weeks of alcohol discontinuation, there was a decline in median fasting glycerol (baseline 50 vs. follow-up 48.5 mmol/L, change − 5.5 mmol/L) and fasting FFA (baseline 0.37 vs. follow-up 0.36 mmol/L, change − 0.05 mmol/L) levels, though these differences were not statistically significant. Moreover, there was no significant difference in change in adipo-IR levels following alcohol discontinuation (median 0.27 vs. 0.23 mmol/L, median change − 0.02 mmol/L).

**Table 2:** Change in metabolic parameters following alcohol cessation (*N* = 24).

Metabolic Parameter	Baseline	Follow-up	Change	*p*-value
Fasting glucose [median (Q1,Q3)] (mg/dL)	94.4 (88.5, 104.4)	97.6 (93.0, 103.0)	2.4 (−3.8, 6.3)	0.13
Fasting insulin [median (Q1,Q3)] (µU/mL)	11.6 (7.6, 18.3)	11.5 (8.8, 18.0)	1.6 (−1.6, 2.3)	0.13
Fasting glycerol [median (Q1,Q3)] (mmol/L)	50.0 (37.0, 58.8)	48.5 (36.8, 60.3)	−5.5 (−9.5, 4.8)	0.33
Fasting FFA [median (Q1,Q3)] (mmol/L)	0.37 (0.29, 0.47)	0.36 (0.24, 0.40)	−0.05 (−0.18, 0.05)	0.59
Adipo-IR [median (Q1,Q3)] (mmol/L)	0.27 (0.13, 0.39)	0.23 (0.11, 0.35)	−0.02 (−0.06, 0.03)	0.40

**Bold** indicates *p* < 0.05.

### Factors associated with change in adipose tissue insulin resistance

We assessed the impact of baseline demographic, behavioral, clinical, and genetic profiles on change in adipo-IR following short-term moderate alcohol discontinuation. On univariable analysis, European ancestry, *CYP2E1* homozygous risk-allele (vs. no mutation), and current smoker (vs. non-smoker) status were significantly associated with change in adipo-IR after short-term alcohol cessation (Table 3). The mean change in adipo-IR following alcohol discontinuation was on average 0.20 mmol/L (95% CI [0.06, 0.33], *p* = 0.006) and 0.14 mmol/L (95% CI [0.003, 0.28], *p* = 0.046) higher for patients with *CYP2E1* homozygous risk-allele (vs. no mutation) and current smoker (vs. non-smoker) status, respectively. Conversely, the mean change in adipo-IR after alcohol discontinuation was on average 0.02 mmol/L lower for each 10-percentage point increase in European ancestry proportion. Compared to HCV- patients, the mean change in adipo-IR was 0.01 mmol/L lower for HCV+ patients but this was not statistically significant (95% CI [−0.12, 0.10], *p* = 0.85).

**Table 3:** Univariable linear regression analyses for adipo-IR *(N* = 24).

Characteristics	Estimate	95% Cl	*p*-value
Age (per decade), years	−0.03	−0.07 to 0.01	0.16
Sex female	−0.06	−0.16 to 0.04	0.25
BMI Overweight/Obese, ref normal	0.05	−0.06 to 0.16	0.32
Waist circumference, cm	0.001	−0.003 to 0.004	0.60
AST (per log2), units/L	−0.03	−0.10 to 0.04	0.36
ALT (per log2), units/L	−0.03	−0.10 to 0.03	0.28
HCV+ status	−0.01	−0.12 to −0.10	0.85
Current smoker	**0.14**	**0.003 to 0.28**	**0.046**
Alcohol consumption duration, years	−0.002	−0.007 to 0.002	0.32
African American ancestry (per 10% points)	0.004	−0.05 to 0.05	0.87
European ancestry (per 10% points)	**−0.02**	**−0.05 to −0.0004**	**0.047**
Native American ancestry (per 10% points)	0.02	−0.01 to 0.04	0.11
PNPLA3, reference (CC)	–	–	–
CG/GG	−0.05	−0.15 to 0.06	0.36
CYP2E1, reference (c1/c1)	–	–	–
Heterozygous (c1/c2)	0.10	−0.06 to 0.26	0.19
Homozygous (c2/c2)	**0.20**	**0.06 to 0.33**	**0.006**

**Bold** indicates *p* < 0.05.

On multivariable forward regression analysis that included HCV status, *CYP2E1* homozygous risk-allele (vs. no mutation) and overweight/obese (defined as BMI ≥ 25) (vs. normal weight) status were significantly associated with change in adipo-IR (Table 4). Following alcohol discontinuation, the mean change in adipo-IR was on average 0.25 mmol/L (95% CI [0.12, 0.38], *p* < 0.001) higher for patients with *CYP2E1* homozygous risk-allele (vs. no mutation), whereas the mean change in adipo-IR was on average 0.13 mmol/L higher for overweight/obese (vs. normal weight) patients (95% CI [0.03, 0.24], *p* = 0.02). As an example, if a patient had HCV+ status and normal weight, our model estimates an average increase of 0.15 mmol/L in adipo-IR in those with *CYP2E1* homozygous risk-allele mutation but a decrease of 0.10 mmol/L in patients without the mutation. If a patient had HCV+ status and no *CYP2E1* mutation, our model estimates an average increase in adipo-IR of 0.03 mmol/L in overweight/obese patients while the average decrease in adipo-IR was 0.10 mmol/L in normal weight patients.

**Table 4:** Multivariable regression analysis of factors associated with adipo-IR following moderate alcohol cessation *(N* = 22).

Characteristics	Estimate	95% Cl	*p*-value
BMI Overweight/Obese, ref normal	**0.13**	**0.03 to 0.24**	**0.02**
HCV+ status	0.05	−0.06 to 0.16	0.37
CYP2E1, ref (c1/c1)	–	–	–
Heterozygous (c1/c2)	0.08	−0.06 to 0.23	0.25
Homozygous (c2/c2)	**0.25**	**0.12 to 0.38**	**< 0.001**

**Bold** indicates *p* < 0.05.

## Discussion

To our knowledge, this is the first prospective study to assess the impact of short-term moderate alcohol discontinuation on adipo-IR using direct measures among a Latino cohort with or without chronic liver disease who underwent comprehensive clinical, ancestral, and genetic profiling. This study is particularly timely given the burden of chronic liver disease in Latino populations and the growing need to understand how optimization of behavioral risk factors influence cardiometabolic health. Overall, there were no significant changes in adipo-IR levels following short-term moderate alcohol discontinuation in our full cohort. In an exploratory analysis, *CYP2E1* homozygous risk-allele and overweight/obesity were independently associated with change in adipo-IR following short-term moderate alcohol discontinuation.

Adipo-IR plays a key role in the development of chronic liver disease through a cascade of events that promote FFA uptake, lipogenesis, and release of pro-inflammatory molecules^23^. While moderate alcohol use has historically been linked to reduced adipo-IR levels^24^, its effect on these metabolic pathways in patients with underlying liver disease remains poorly understood. Although our study does not specifically focus on liver-related outcomes, both alcohol use and adipo-IR are significant contributors to the progression of chronic liver disease^23,25^. In the context of liver disease with known adverse metabolic consequences, such as HCV or steatotic liver disease, understanding the interplay between metabolic dysfunction, such as adipo-IR, and modifiable risk factors (e.g., alcohol cessation, HCV infection) is of growing interest to the field.

We identified overweight/obese status as an independent factor associated with greater change in adipo-IR levels following short-term alcohol discontinuation. Excessive visceral adiposity promotes the release of adipokines, proinflammatory factors, and FFAs, all of which contribute to impaired insulin signaling^26^. Our previous work has also demonstrated that adipo-IR is linked to dyslipidemia, specifically elevated small dense LDL^27^, along with several other cardiovascular-related outcomes^28^. Interestingly, alcohol discontinuation led to more favorable improvements in adipo-IR among normal-weight participants than those with overweight or obesity, suggesting that adiposity is the primary driver of adipo-IR. This association was evident even in the presence of HCV, a chronic liver disease known to cause metabolic dysfunction. While this study was conducted prior to the era of highly effective direct-acting antiviral (DAA) therapies, these findings remain relevant in the contemporary HCV cohorts. Firstly, substantial gaps in the HCV care cascade leave a notable proportion of Latino patients untreated^29,30^. Moreover, though metabolic abnormlities tend to improve, HCV cure may not fully reverse underlying metabolic dysfunction^31^. Therefore, in addition to HCV treatment and alcohol discontinuation, lifestyle modification with weight loss remains a critical component of management of metabolic dysfunction-associated chronic liver disease.

In an exploratory analysis, we observed an association between *CYP2E1* RsaI homozygous polymorphism [c2/c2] and changes in adipo-IR following alcohol discontinuation. The proportion of *CYP2E1* c2/c2 frequency in this cohort was 14% which is similar to that reported in the other Latino cohorts at 16%^10^. This mutant allele, more prevalent in Latino populations, enhances the expression of *CYP2E1*, a cytochrome P450 family enzyme known for its metabolism of alcohol and several other substrates^10,32^. Known for its implication in ALD, alcohol consumption induces *CYP2E1* expression leading to the production of reactive oxygen species, which promotes cellular damage, lipid peroxidation, and mitochondrial injury^32^. *CYP2E1* also plays a central role in driving metabolic dysfunction-associated steatotic liver disease through two principal pathways. Notably, *CYP2E1* contributes to the metabolism of polyunsaturated fatty acids into toxic ω-hydroxylated acids, driving progression of liver disease from steatosis to steatohepatitis^33^. Secondly, *CYP2E1* expression has been linked to the development of adipo-IR. Mice model studies have shown that knocking out the *CYP2E1* gene confers protection against IR^34^, whereas its overproduction results in IR, hepatic steatosis, and liver damage^35^. While evidence in human models is limited^36,37^, our findings add preliminary evidence that *CYP2E1* homozygosity may be associated with changes in adipo-IR following alcohol discontinuation.

While we are now firmly in the era of highly effective DAA therapies, significant real-world clinical gaps in HCV eradication persist, and a notable proportion of vulnerable populations remain untreated; similarly, treatment for concurrent alcohol use disorder remains vastly suboptimal in individuals with chronic liver disease. Furthermore, emerging data show that even when HCV cure and alcohol abstinence are successfully achieved, viral eradication and sobriety do not fully reverse underlying metabolic dysfunction—particularly among individuals with pre-existing risk factors like obesity and Latinx ethnicity^38^.

The major strength of our study was the use of direct measurements for adipo-IR along with a comprehensive assessment of demographic, clinical, and genetic profiles. There were some limitations to our study. Although our sample size was limited, it is comparable to several other studies assessing metabolic profiles using direct measures^9,37^, given the logistical challenges of performing a 2-day inpatient evaluation with a larger sample size. Alcohol intake was assessed via self-report, which is susceptible to underreporting and biases, such as recall error and social desirability^39,40^. However, random biomarkers of alcohol use were also used to confirm maintenance of alcohol cessation during follow-up. This study did not evaluate the impact of long-term alcohol cessation. However, the 6-week period was intentionally chosen to ensure complete alcohol cessation and to minimize confounding factors that may arise during longer-term follow-up.

In summary, although short-term moderate alcohol discontinuation was not associated with a significant overall change in adipo-IR, obesity status and the presence of the *CYP2E1* polymorphism appear to influence adipo-IR change following alcohol cessation in this Latino cohort. These hypothesis-generating findings suggest that further research may be needed to elucidate the underlying interaction between *CYP2E1* and adipo-IR and explore the broader clinical implications of these findings in a larger cohort and within the context of long-term alcohol discontinuation.

## Data Availability

All relevant data is shared in the manuscript. No additional data are available.

## Abbreviations

ALD: Alcohol-associated liver disease
HCV: Hepatitis C virus
IR: Insulin resistance
adipo-IR: Adipose tissue insulin resistance
OGTT: Oral glucose tolerance test
BMI: Body mass index
IRB: Institutional review board
STROBE: Strengthening the reporting of observational studies in epidemiology
IST: Insulin suppression test
FFA: Free fatty acids
CYP2E1: Cytochrome P450 2E1
AIMs: Ancestry informative markers
ALT: Alanine transaminase
AST: Aspartate aminotransferase
LDL: Low-density lipoprotein
DAA: Direct-acting antiviral
